# Altered Sense of Body Ownership and Agency in Posttraumatic Stress Disorder and Its Dissociative Subtype: A Rubber Hand Illusion Study

**DOI:** 10.3389/fnhum.2018.00163

**Published:** 2018-05-01

**Authors:** Daniela Rabellino, Dalila Burin, Sherain Harricharan, Chantelle Lloyd, Paul A. Frewen, Margaret C. McKinnon, Ruth A. Lanius

**Affiliations:** ^1^Department of Psychiatry, University of Western Ontario, London, ON, Canada; ^2^Spatial, Motor & Bodily Awareness, Research Group, Psychology Department, University of Turin, Turin, Italy; ^3^Smart-Aging Research Center & IDA, Institute of Development, Aging and Cancer, Tohoku University, Sendai, Japan; ^4^Department of Neuroscience, University of Western Ontario, London, ON, Canada; ^5^Departments of Psychiatry and Behavioural Neurosciences, McMaster University, Hamilton, ON, Canada; ^6^Department of Psychology, University of Western Ontario, London, ON, Canada; ^7^Mood Disorders Program, St. Joseph's Healthcare, Hamilton, ON, Canada; ^8^Homewood Research Institute, Guelph, ON, Canada; ^9^Department of Medical Imaging, Lawson Health Research Institute, London, ON, Canada

**Keywords:** body ownership, sense of agency, PTSD, dissociation, rubber hand illusion

## Abstract

Traumatic experiences have been linked to the development of altered states of consciousness affecting bodily perception, including alterations in body ownership and in sense of agency, the conscious experience of the body as one's own and under voluntary control. Severe psychological trauma and prolonged distress may lead to posttraumatic stress disorder (PTSD). Together, symptoms of derealization and, related specifically to the sense of body ownership and agency, of depersonalization (where parts of the body or the entire body itself is perceived as detached and out of control), constitute the dissociative subtype (PTSD+DS). In this study, we explored the Rubber Hand Illusion, an experimental paradigm utilized to manipulate sense of body ownership in PTSD (*n* = 4) and PTSD+DS (*n* = 6) as compared to healthy controls (*n* = 7). Perceived finger location and self-report questionnaires were used as behavioral and subjective measures of the illusion, respectively. In addition, the correlation between the illusion's effect and sense of agency as a continuous feeling of controlling one's own body movements was explored. Here, a lower illusion effect was observed in the PTSD as compared to the control group after synchronous stimulation for both the proprioceptive drift and subjectively perceived illusion. Moreover, by both proprioceptive drift and by subjective ratings, the PTSD+DS group showed a response characterized by high variance, ranging from a very strong to a very weak effect of the illusion. Finally, sense of agency showed a trend toward a negative correlation with the strength of the illusion as subjectively perceived by participants with PTSD and PTSD+DS. These findings suggest individuals with PTSD may, at times, maintain a rigid representation of the body as an avoidance strategy, with top-down cognitive processes weakening the impact of manipulation of body ownership. By contrast, the response elicited in PTSD+DS appeared to be driven by either an increased vulnerability to manipulation of embodiment or by a dominant top-down cognitive representation of the body, with disruption of multisensory integration processes likely in both cases. Taken together, these findings further our understanding of bodily consciousness in PTSD and its dissociative subtype and highlight the supportive role played by sense of agency for the maintenance of body ownership.

## Introduction

Conscious experience of the self is a complex phenomenon that assumes a bodily representation of the self, including thoughts, feelings, and actions (Damasio, 1994; Gallagher, 2000, 2013). Here, the representation of the body in humans has been described as multidimensional, involving both “bottom-up” multiple sensory inputs (vision, touch, and proprioception) and “top-down” conceptual representations of the body (Tsakiris and Haggard, 2005; Longo et al., 2008). The integration of low-level sensorimotor processes coherent with top-down meta-representations of the body is thought to lead an individual to experience a comprehensive and ongoing bodily experience (Balconi, 2010) and has been associated with body ownership, the sense of the body as belonging to the self (Ghallager, 2000; Tsakiris, 2010). Moreover, the self-consciousness of having/owning a body comprises not only a sense of ownership but also the sense of agency over one's own body, two components crucial to building a sense of bodily self-consciousness (Longo et al., 2008; Serino et al., 2013). Critically, embodiment, the self-awareness of being located in one's own body (Longo et al., 2008; Lewis and Lloyd, 2010), has been described as compromised in both neurological and psychiatric conditions (Kenna and Sedman, 1965; Alper et al., 1997; Baker et al., 2013), including in posttraumatic stress disorder (PTSD; Ataria, 2015; Frewen and Lanius, 2015; Lanius, 2015; Rabellino et al., 2016), a psychiatric disorder following the experience of severe and/or multiple psychological trauma.

PTSD includes re-experiencing, avoidance, hyperarousal symptoms, and negative alterations in mood and cognition, symptoms that often have bodily manifestations, including bodily distress and activation of bodily defensive actions such as fight-and-flight responses. These symptoms suggest a link between high-level and low-level cognitive/sensory mechanisms. Bottom-up multisensory processing is thought to be particularly involved during re-experiencing and hyper-arousal symptoms, where the traumatic event is relived as if it were re-occurring at the present moment with concurrent bodily perceptions and reactions. By contrast, avoidance symptoms have been linked to a top-down over-modulation of emotional reaction and to a coping style characterized by active avoidance of trauma reminders and potential triggers, as well as emotional detachment/restricted affect (such as emotional numbing, often associated with alexithymia; Frewen et al., 2008, 2012; Lanius et al., 2010; APA, 2013).

Critically, a dissociative subtype of PTSD (PTSD+DS) has been added recently to the DSM-5 (APA, 2013) and is characterized by derealization (feeling that one's external surroundings are unreal, dreamlike, or distorted) and depersonalization, symptoms that target specifically alterations in body ownership (feeling detached from the body and that part of or the whole body is not one's own; Lanius et al., 2012; Spiegel et al., 2013). Depersonalization symptoms in PTSD may represent a unique opportunity to investigate the phenomenology of body ownership and agency, where the detachment from one's own body leads the individual to experience a failure of the perceptual integrated self (Spiegel et al., 2013). Depersonalization symptoms can be characterized by partial disembodiment (or partial loss of body ownership), where part of the body (e.g., a hand or a foot) is experienced as “non-self,” namely alien, not one's own, as well as by full disembodiment (or complete loss of body ownership), where the whole body is felt as “non-self” (Sierra and David, 2011; Frewen and Lanius, 2015).

Furthermore, individuals that develop PTSD may experience a weakened sense of agency (Ataria, 2015), where impairment of intentional control over one's own movements occurs, with consequent feelings of helplessness. During trauma, the loss of agency can be experienced as loss of control because someone else is controlling the subject's body movements (e.g., in the case of rape or torture). In addition, an extreme loss of agency can be experienced when an individual feels unable to voluntarily control his/her own body as occurs, for example, during freezing states (Herman, 1992; Ataria, 2015). During freezing states, which can be a form of death feigning in order to protect the individual from a predator during the traumatic event, the individual experiences extreme fear, muscle tension, and an inability to move, with dual activation of the sympathetic and parasympathetic nervous system (Schauer and Elbert, 2010; Kozlowska et al., 2015).

As described above, sense of body ownership and sense of agency represent two dissociable aspects of embodiment and self-consciousness (Tsakiris et al., 2007; Balconi, 2010; Kalckert and Ehrsson, 2012) that also appear to share a close interaction. Indeed, both afferent peripheral signals and efferent bodily movements contribute to bodily ownership, as agency has been proposed to contribute to building the sense of bodily ownership (Tsakiris et al., 2006). Their reciprocal interplay, however, is yet to be completely understood (Dummer et al., 2009; Kalckert and Ehrsson, 2012; Burin et al., 2015).

Given the relation between PTSD symptomatology and disturbances in bodily self-consciousness, the objective of the current study was to explore the phenomenology of body ownership alterations and loss of agency via the Rubber Hand Illusion (RHI) paradigm. The RHI manipulates visual, tactile, and proprioceptive inputs from one's hidden hand through synchronous brushing of the hidden real hand and a plausible visible rubber hand. The illusion temporarily alters the sense of ownership of the hidden hand, where the rubber hand seems to substitute for the real hand in the subjective representation of the body (Botvinick and Cohen, 1998; Ehrsson et al., 2004). The RHI procedure therefore provides an ideal paradigm for the manipulation and investigation of body ownership and its alterations in individuals with dissociative symptoms. A case study with participants diagnosed with the dissociative subtype of PTSD showed a strong illusion effect of the RHI task, with associated alterations in body ownership and temporary exacerbation of depersonalization and derealization during the task (Rabellino et al., 2016). Critically, however, investigations comparing PTSD with and without the dissociative subtype and healthy controls are currently lacking.

We hypothesized that PTSD individuals with the dissociative subtype (PTSD+DS) would show a significantly stronger effect of the illusion, with relative alterations in the sense of body ownership as compared to healthy controls. We were also interested in exploring possible differences in the effects on body ownership between the two PTSD groups (PTSD vs. PTSD+DS). Here, whereas avoidance symptoms characterizing the PTSD group were expected to induce a more rigid top-down representation of the body aimed at avoiding any possible manipulation (a potential trigger), depersonalization symptoms characterizing the PTSD+DS group were expected to result in flexible and more easily alterable body representation (Sierra and David, 2011; Frewen and Lanius, 2014, 2015) linked to a stronger illusion effect of the RHI. Moreover, we hypothesized that an initial weaker sense of agency, here interpreted as the continuous feeling that one's own body is under one's own control (Haggard and Chambon, 2012), would show a significant correlation with the strength of the illusion induced by the RHI paradigm in individuals with PTSD. In summary, our study objectives were to investigate: (a) body ownership in PTSD during the RHI; (b) body ownership in the dissociative subtype of PTSD during the RHI; and (c) the correlation between body ownership and sense of agency in the whole PTSD sample.

## Materials and methods

### Participants

Four participants with a primary diagnosis of PTSD (PTSD) and six participants with a diagnosis of PTSD with the dissociative subtype (PTSD+DS) were enrolled through community advertisement in London, ON, Canada. Seven healthy controls (HC) were enrolled through community advertisement in Turin, Italy. Whereas the Structured Clinical Interview for DSM-IV Axis I disorders [SCID-I; First et al., 2000; 2002 (Italian edition)] was administered to assess the participants for psychiatric disorders, PTSD symptom severity was assessed through the Clinician Administered PTSD Scale for DSM-4 and for DSM-5^1^ (CAPS-4, Blake et al., 1995; CAPS-5, Weathers et al., 2013; cut-off score ≥50 for CAPS-4 and criteria met for CAPS-5). The two CAPS items on depersonalization (measuring persistent or recurrent experience of feeling detached from one's mental processes or body) and derealization (measuring persistent or recurrent experience of unreality of surroundings) were utilized to identify PTSD participants that met criteria for the dissociative subtype (on CAPS-4 individual item scores range is 0–4 on two separate subscales—frequency of the experience and intensity of the experience -; when the total score frequency + intensity ≥4, the participant was included in the dissociative subtype of PTSD group; on CAPS-5 individual item scores range is 0–4 on a single scale, when the score was ≥2, the participant was included in the dissociative subtype of PTSD group) as per standard methods (Weathers et al., 1999; Rabellino et al., 2015). Dissociative symptomatology was further assessed with the Multiscale Dissociation Inventory (MDI; Briere, 2002), a 30-item self-report questionnaire that measures dissociative symptoms (disengagement, depersonalization, derealization, emotional constriction, memory disturbance, and identity dissociation), and the Structured Clinical Interview for Dissociative Disorders (SCID-D; Steinberg et al., 1993), designed specifically to assess dissociative symptomatology (depersonalization, derealization, and amnesia). Participants with a diagnosis of psychosis, bipolar disorder, significant medical or neurologic conditions, history of head injury (loss of consciousness), and/or alcohol or substance abuse not in sustained full remission were excluded. HC had no current history of psychiatric disorder or neurological injury. All participants were right-handed (Oldfield, 1971). The study was approved by the Health Sciences Research Ethics Board of Western University, Canada, and by the local Bioethical Committee from University of Turin, Italy, and all participants provided informed written consent.

### Materials

**Figure 1:**
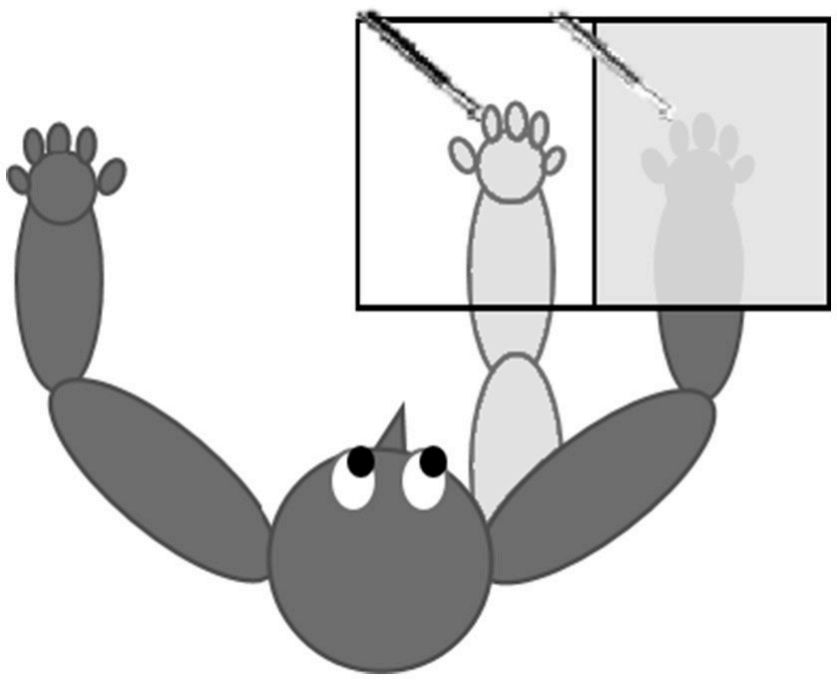
Setting of the RHI procedure. The participant is seated with both hands (dark gray) placed on a table; the real right hand is inside a covered box that prevents visual feedback of the real right hand. The rubber hand (light gray) is aligned with participant's shoulder and is placed visibly in the opened part of the box. Both the rubber hand's and the real hand's index fingers are stimulated synchronously or asynchronously.

As described in Rabellino et al. (2016) and following standard procedures (Burin et al., 2015), the RHI task was performed using a black box (60 × 40 × 20 cm) divided in half (30 × 40 × 20 cm) by a perpendicular panel. One half of the box (where the participants inserted their own hand) was covered in order to hide the participant's real hand from view and to allow a view of the life-like rubber hand (positioned in the other half) only. Participants wore a cape to ensure that only the rubber hand was in view, while the alignment of the rubber hand with the participant's shoulder rendered the rubber hand position plausible, closer to the body midline with respect to the real participant's hand. Either the real or the rubber hand were positioned to point fingers forward and to face palms down, while an approximate distance of 15 cm separated the real and the rubber hand. An illustration of the setting is provided in Figure 1.

The procedure consisted of brushing synchronously vs. asynchronously the index finger of the rubber hand and the real dominant hand (right hand for all participants) during two consecutive 2-minute trials (Costantini and Haggard, 2007; Ocklenburg et al., 2011). The trial order (synchronous vs. asynchronous) was counterbalanced between participants.

### Procedure

#### Proprioceptive drift

Prior to each trial, a flat lid was used to cover the box. After placing a soft ruler (in centimeters) upon the lid, the participant was asked to report the number on the ruler that corresponded to her/his perceived index finger's location (Burin et al., 2015). This inquiry was repeated six times with a random change of the ruler's position each time, in order to prevent the participants from repeating their previous answers. The same procedure was applied after each trial (synchronous and asynchronous) to determine proprioceptive drift, obtained by subtracting the average post-trial estimations from the average pre-trial estimations for each subject (Botvinick and Cohen, 1998). Proprioceptive drift, the perception that one's own hand is closer to the rubber hand after the illusion procedure, is considered a reliable and objective measure of the illusion effect (Longo et al., 2008; Ehrsson, 2012), particularly when confirmed by subjective measures of the illusion (see questionnaires presented below). Although there is no standard cut-off measure of proprioceptive drift in healthy subjects for the illusion to be considered effective, previous studies have documented a drift that ranged from 0.76 cm (Longo et al., 2008) to 9 cm (Botvinick and Cohen, 1998) when the illusion effect took place.

#### Questionnaires

The nine-item questionnaire created by Botvinick and Cohen (1998; here referred to as RHI Likert scale) was administered verbally to assess subjective perception of the illusion after each trial. Scores ranged from −3 indicating complete disagreement to +3 indicating complete agreement. Whereas the first three questions consist of target questions relative to the illusion effect, the remaining questions are administered to control for task compliance (see Supplementary Material S1). Items were administered in a random order for each trial and for each participant.

A questionnaire, here named the Sense of Agency questionnaire, investigated the sense of agency as the general and usual feeling of having intentional control over movements acted by one's own body (considering the last month; see Supplementary Material S2) and was administered prior to the experiment. Items of the Sense of Agency questionnaire were adapted from a previous study by Kalckert and Ehrsson (2012) and included six items (3 target and 3 control items) scored on the same scale used for the RHI Likert scale (see Supplementary Material S2 for details). Items were administered in a random order for each participant.

At the end of the experimental session, participants were encouraged to describe sensations and feelings during the experiment. Indeed, the phenomenology of the response to the RHI paradigm represents a unique opportunity for the understanding of the dissociative subtype of PTSD, where dissociative states can affect the perception of one's own body and its relationship with the surrounding world.

### Data analyses

Statistical analyses were performed using SPSS v24 (IBM Corporation). Descriptive analyses were initially conducted to explore the distribution of the data (Shapiro-Wilk to test normality) and the homogeneity of the variance (Levene's test). Averages on psychological and demographic data, as well as between-group comparisons (one-way ANOVA) were then computed.

In order to compare the effect of synchronous vs. asynchronous trial on drift (post- pre-measurements) in the whole sample, a Wilcoxon signed ranks test (2-tailed) was conducted collapsing the groups in one sample. We then focused on the effect of drift after synchronous trials by performing a one-way Welch's ANOVA (three groups: CNTR, PTSD, PTSD+DS), a test taking into account unequal sample sizes and inhomogeneity of the variance, followed by *post-hoc* Games-Howell between-group comparisons. Additional comparisons were run for the PTSD+DS group to explore the high variance observed as compared to the PTSD and the control group (Levene's test).

With regard to the subjective effect of the illusion, as measured by the RHI Likert scale, ratings for each item were standardized by means of an ipsatization procedure (to control for response bias; Romano et al., 2014; Burin et al., 2015). Due to the nature of the data (non-normal distribution), we compared the subjective ratings during synchronous vs. asynchronous trial through a Wilcoxon non-parametric test within the whole sample (CNTR, PTSD, PTSD+DS) on each of the target items (first three items: Q1, Q2, Q3, here referred as “real” items), the average of the real items as well as the real items after subtraction of the control items (real – control items). Subsequently, we conducted Kruskal-Wallis H tests (3 groups: CNTR, PTSD, PTSD+DS) to investigate between-group differences on the subjective ratings after synchronous trials for the first three items (Q1, Q2, Q3, here referred as “real” items), the average of the real items as well as the real – control items. As the PTSD+DS group showed a significantly higher variance than the other groups (Levene's test), we explored between-group comparisons on the subjective ratings in the PTSD group as compared to the control group after synchronous trials. Here, Mann-Whitney tests were performed on each item, as well as on the real items collapsed and the real – control items. Again, the high degree of variance present in the PTSD+DS group was compared to the other groups' variance at each item of the RHI Likert scale.

Correlations (Spearman's rho, two-tailed) between either drift measurements and RHI Likert scale ratings (average of the first three items, here referred as “real” items) and the sense of agency ratings (average of the first three items, here referred as “real” items) within the whole PTSD sample (PTSD and PTSD+DS) were performed.

Finally, direct reports from participants were categorized into themes using qualitative methods (Boyatzis, 1998; Braun and Clarke, 2006).

## Results

### Participants

No significant group differences were found relative to age, sex, or education. See Table 1 for a complete report on psychological and demographic characteristics.

**Table 1:** Demographic and psychological characteristics.

**Clinical and demographical characteristics**	**HC (*n* = 7)**	**PTSD (*n* = 4)**	**PTSD+DS (*n* = 6)**	**ANOVA/ttest/χ2 (*p*)**
Age (mean ± SD) years	41.86 ± 9.68	38 ± 9.57	51.17 ± 9.35	0.105
Gender (F) frequency	6	3	5	0.902
Education	16.42 ± 3.73	16.5 ± 1	16.6 ± 2.3	0.989
CAPS tot score (mean ± SD)	5.28 ± 9.14 (CAPS-4)	33.25 ± 8.8 (CAPS-5)	63 ± 7.07 (CAPS-4) 46.5 ± 10.34 (CAPS-5)	0.100 (CAPS-5)^*^
MDI tot score (mean ± SD)	N/A	40.75 ± 4.11	93.33 ± 35.34	0.014
SCID_D comorbidity frequency	N/A	–	DDNOS (2) DDNOS-in partial remission (4)	–
SCID_I comorbidity (current [past]) frequency	Adjustment disorder [2]	Major depressive disorder (1 [2])	Major depressive disorder (3 [3])	–
		Major Depressive Episode [1]	Panic disorder with agoraphobia [1]	
		Lifetime history of alcohol abuse or dependence [2]	Obsessive-compulsive disorder [1]	
			Eating disorders (1)	
			Somatoform disorder [2]	
			Lifetime history of alcohol abuse or dependence [3]	
			Lifetime history of substance abuse or dependence [3]	

*CAPS, Clinician Administered PTSD Scale; DDNOS, Dissociative Disorder Not Otherwise Specified; HC, healthy controls; MDI, Multiscale Dissociation Inventory; PTSD, posttraumatic stress disorder group; PTSD+DS, dissociative subtype of the posttraumatic stress disorder group; SCID-D, Structured Clinical Interview for Dissociative Disorders; SCID-I, Structured Clinical Interview for DSM-IV Axis I disorders*.

* *t-test between PTSD and PTSD+DS based on CAPS-5*.

### Proprioceptive drift

Data were normally distributed for drift during the synchronous condition and non-normally distributed during the asynchronous condition.

#### Synchronous vs. asynchronous trials

A comparison between trials (synchronous-SYN vs. asynchronous-ASYN) showed a trend toward a significantly higher drift during the SYN as compared to the ASYN condition within the whole sample (SYN>ASYN: Z = 1.752, *p* = 0.080, *r* = −0.425; see Table 2).

**Table 2:** Proprioceptive drift.

**SYN vs. ASYN (Wilcoxon signed-rank test)**	**Z**	***p***
Collapsed across groups	−1.752	0.080
**post- pre-SYN**	**Welch test**	***p***
One-way Welch's ANOVA	4.853	0.041^*^
CNTR>PTSD (Games-Howell *post-hoc*)	2.256 (mean difference)	0.036^*^
**Homogeneity of the variance post- pre-SYN (Levene's test)**	**Levene stat**	***p***
CNTR vs. PTSD+DS	9.410	0.011^*^
PTSD vs. PTSD+DS	5.633	0.045^*^
PTSD vs. CNTR	0.491	0.591

*ASYN, asynchronous condition; CNTR, control group, PTSD; posttraumatic stress disorder group; PTSD+DS, dissociative subtype of PTSD; SYN, synchronous condition*.

* *denotes p < 0.05*.

#### Between-groups results for post- pre-synchronous trials

Significant results emerged from the one-way Welch's ANOVA [*F*_(2, 14)_ = 4.853, *p* = 0.041].

#### Post- pre-synchronous trials in PTSD vs. HC

**Figure 2:**
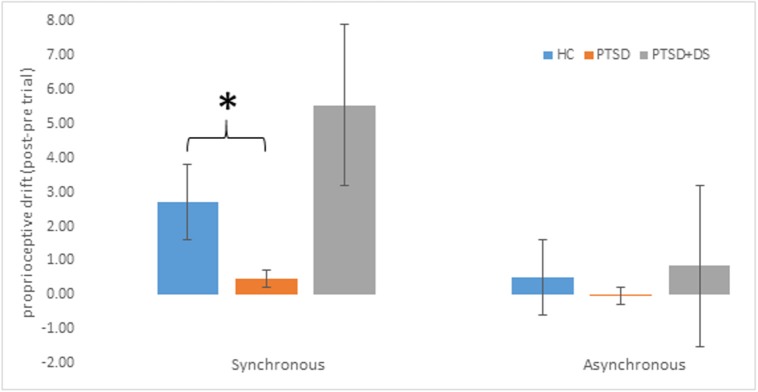
Proprioceptive drift (averaged post-pre- trial estimation) in the three groups. Positive scores refer to perceived location closer to the rubber hand (in cm). Error bars represent standard errors. Asterisks indicate significant comparisons (^*^p < 0.05). HC: healthy controls, PTSD: posttraumatic stress disorder group, PTSD+DS: dissociative subtype of the posttraumatic stress disorder group.

With respect to the proprioceptive drift during the SYN condition only, *post-hoc* tests showed that the PTSD group had a significantly *lower* illusion effect as compared to the control group (CNTR: M = 2.71 ± 1.36 cm, PTSD: M = 0.46 ± 1.04 cm; CNTR > PTSD: *p* = 0.036; see Table 2). No other significant differences were found between groups (all *p* > 0. 469). Proprioceptive drifts during synchronous and asynchronous trials are illustrated in Figure 2.

#### Levene's tests on the homogeneity of the variance in post- pre-synchronous trials

The PTSD+DS group showed a grossly higher variance in the drift measurements (variance: SYN = 95.642, ASYN = 24.335) as compared to the PTSD group (variance: SYN = 1.081, ASYN = 0.951) and the HC (variance: SYN = 1.849, ASYN = 7.791). These results were supported by the Levene's test of the homogeneity of variance for the post- pre-SYN condition (CNTR vs. PTSD+DS: Levene stat = 9.410, *p* = 0.011; PTSD vs. PTSD+DS: Levene stat = 5.633, *p* = 0.045; PTSD vs. CNTR: Levene stat = 0.491, *p* = 0.501; see Table 2).

### Subjective ratings

#### Synchronous vs. asynchronous

As compared to the ASYN condition, the SYN condition showed a significantly higher perception of the illusion using all of the real items of the RHI Likert scale (Q1, Q2, Q3), considered either separately or as an average (SYN > ASYN Q1: Z = 2.816, *p* = 0.005, Q2: Z = 2.599, *p* = 0.009; Q3: Z = 2.047, *p* = 0.041, average of the real items: Z = 2.921, *p* = 0.003), as well as for the real—control items (SYN > ASYN: Z = 2.586, *p* = 0.010; see Table 3) within the whole sample.

**Table 3:** Subjective ratings.

	**Q1**		**Q2**		**Q3**		**Average real Q**		**Average real–control Q**	
**SYN > ASYN (Wilcoxon signed-rank test)**	**Z**	***p***	**Z**	***p***	**Z**	***p***	**Z**	***p***	**Z**	***p***
Collapsed across groups	2.816	0.005^*^	2.599	0.009^*^	2.047	0.041^*^	2.921	0.003^*^	2.586	0.01^*^
**SYN (Mann-Whitney test)**	**U**	***p***	**U**	***p***	**U**	***p***	**U**	***p***	**U**	***p***
CNTR>PTSD	13	0.850	1	0.014^*^	5	0.089	2	0.023^*^	3	0.037^*^
**Homogeneity of the variance SYN (Levene's test)**	**Levene stat**	***p***	**Levene stat**	***p***	**Levene stat**	***p***	**Levene stat**	***p***	**Levene stat**	***p***
CNTR vs. PTSD+DS	1.163	0.304	6.457	0.027^*^	1.189	0.299	1.245	0.288	1.225	0.292
PTSD vs. PTSD+DS	0.070	0.798	5.54	0.046^*^	6.28	0.037^*^	0.193	0.672	0.605	0.459

*ASYN, asynchronous condition; CNTR, control group; PTSD, posttraumatic stress disorder group; PTSD+DS, dissociative subtype of PTSD; Q, question item; SYN, synchronous condition*.

* *denotes p < 0.05*.

#### Between-groups results for synchronous trials

No significant between-group results emerged when considering scores on Q1 (χ^2^ = 1.018, *p* = 0.601), Q3 (χ^2^ = 3.721, *p* = 0.156), and the real—control items (χ^2^ = 4.251, *p* = 0.119). However, between-group results showed a trend toward significance when considering scores on Q2 (χ^2^ = 5.708, *p* = 0.058, η^2^ = 0.131) and the average of the real items (χ^2^ = 5.032, *p* = 0.081, η^2^ = 0.079).

#### Synchronous trials in PTSD vs. HC

**Figure 3:**
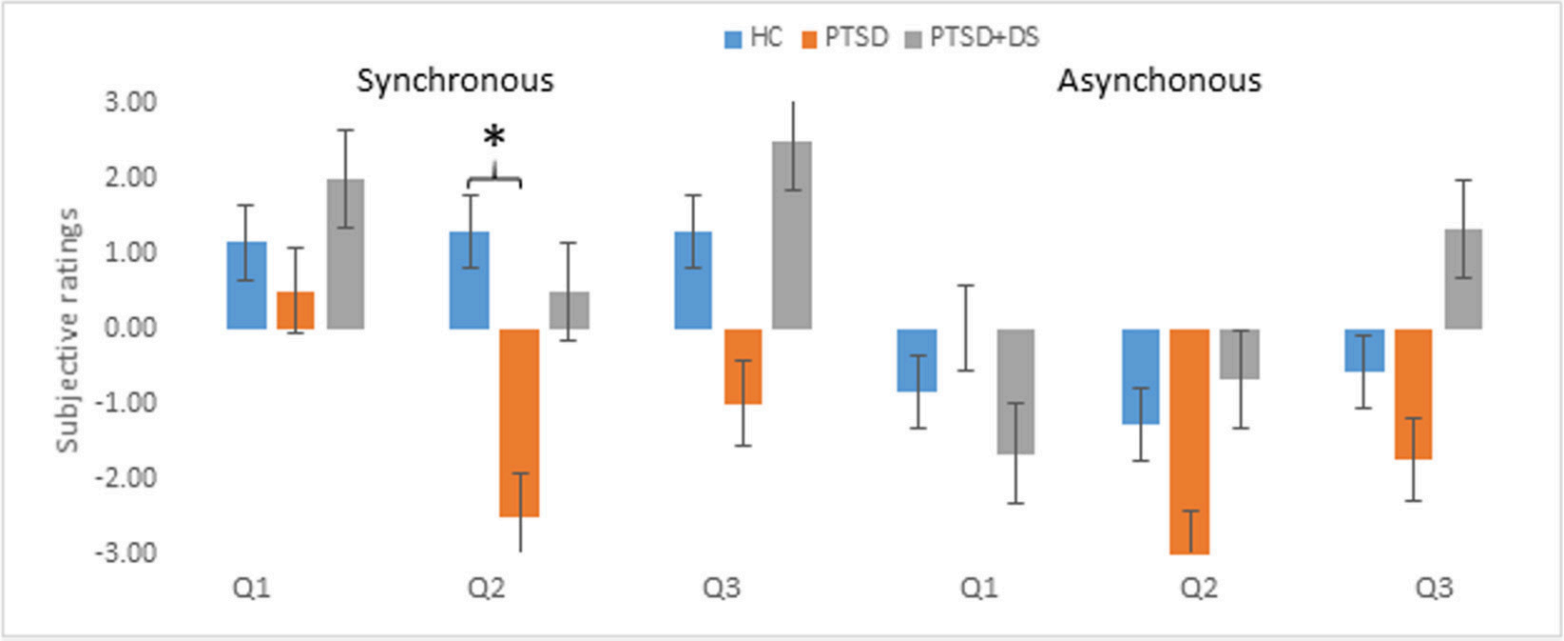
Subjective ratings of RHI in the three groups. Positive scores refer to the subjective experience of the illusion. Error bars represent standard errors. Asterisks indicate significant comparisons (*p < 0.05). HC, healthy controls; PTSD, posttraumatic stress disorder group; PTSD+DS, dissociative subtype of the posttraumatic stress disorder group.

Comparing PTSD to HC, the PTSD group showed a significantly lower perception of the illusion during the SYN condition for item Q2(U = 1, *p* = 0.014). Similarly, as compared to HC, the PTSD group showed a lower illusion perception during the SYN condition for the average of the real items (U = 2, *p* = 0.023), and for the real—control items (U = 3, *p* = 0.037; see Table 2; Figure 3). Considered separately, items Q1 and Q3 did not show any significant difference between groups (see Supplementary Material S2 for items Q1, Q2, and Q3).

#### Levene's tests on the homogeneity of the variance in synchronous trials

The PTSD+DS again showed a significantly higher variance for Q2 as compared to the control group (Levene stat = 6.457, *p* = 0.027), and for Q2 (Levene stat = 5.54, *p* = 0.046) and Q3 (Levene stat = 6.28, *p* = 0.037) as compared to the PTSD group. No significant differences in variance emerged in the PTSD vs. the control group (see Table 3).

### Sense of agency

**Figure 4:**
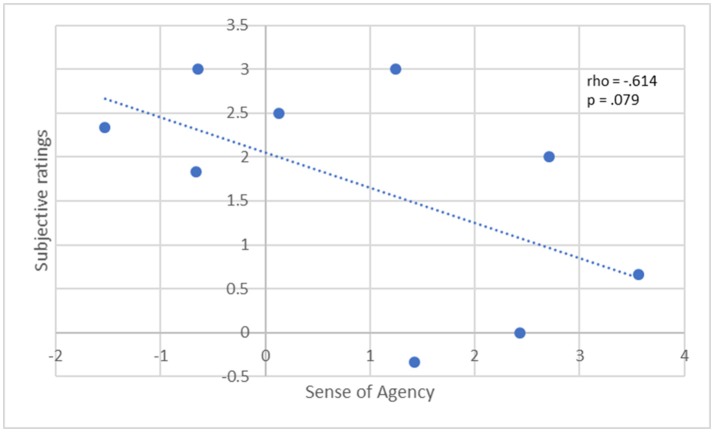
Correlation between subjective ratings of RHI (average real Q) and Sense of Agency (average real items) in the whole PTSD group (PTSD and PTSD+DS). The dotted line depicts a negative correlation; Spearman's rho and *p*-value are reported at the top right. Sense of Agency scores are missing for one subject.

Subjective ratings on the illusion perception showed a trend toward a significant negative correlation with the sense of agency (rho = −0.614, *p* = 0.079) after synchronous trials (see Figure 4). No other significant correlations emerged with the sense of agency.

### Phenomenology

Following drift measurements and the administration of questionnaires, PTSD participants were prompted to describe their subjective experience of the experiment (detailed reports of three of the PTSD+DS participants can be found in Rabellino et al., 2016).

#### Coping strategies in PTSD+DS

The diverse experiences reported by PTSD+DS participants (here referred to as P1, P2, P3, etc. for convenience) in response to the RHI followed three distinct patterns: (a) perceiving that one's own hidden hand is moving back and forth between the real hand's and the rubber hand's position; (b) feeling that one's own hand has jumped through the box division and become one with the rubber hand; (c) feeling that one's own hand is located somewhere in the space between the two hands. Individual reports detailing each coping strategy are described below:

- *Perceiving that one's own hidden hand is moving back and forth between the real hand's and the rubber hand's position*. This experience was described by one PTSD+DS participant as: P4 “It feels like it [index finger] is going between numbers [referring to the drift measurements], like it's going back and forth, back and forth.” Another participant reported the following after asynchronous brushing: P5 “It seems like the hand is going back and forth, almost jumping like the brushing,” and during the synchronous condition: “The hand was going back and forth, afterwards my hand was where the rubber hand was” (this participant showed a drift after SYN = 10.5 cm, and after ASYN = 3.3 cm, while the subjective perception of the illusion after SYN = −0.06, and after ASYN = −1.06 using the average score for the real items);
- *Feeling that one's own hand has jumped through the box division and become one with the rubber hand*. This experience was reported by a participant showing a strong illusion effect (drift after SYN = 22.83 cm, drift after ASYN = 1.17 cm; subjective ratings after SYN = 2.3, after ASYN = −0.03 using the average score for the real items) in these terms: P1 “it didn't drift it flew […] it's like it jumped […] once it jumped over it was there, there was no more coming back” (also reported in (Rabellino et al., 2016));
- *Feeling that one's own hand is located somewhere in the space between the two hands*. For example, one participant described this experience as being like a non-specific location of one's own hand in space: P6 “I felt the sensation but there was no location to it. […] I was looking at the hand, and the feeling was somewhere off in space […] You can see in your body where it's happening …but it's not really happening there, just hanging off in space somewhere.”

#### Top-down body representation vs. bottom-up sensory processing

PTSD+DS participants described the conflict occurring between cognitive representation of their own body and the incoming sensory information as follows: P5 “Logically, I knew [it couldn't be], but it felt like in front of me; as the measurements went on, it felt like it went back where it should be,” P2 “Because it is a disconnect, it's not mine and I should be able to figure that out but it wasn't … near the end I wasn't sure and so that was difficult,” P1 “… knowing that it should be there but this is where it feels like … and so I was really having problems at that point with a number “cause I could intellectualize it but that's not what it was feeling like.” These feelings appeared to trigger a familiar sensation of uncertainty with respect to their own beliefs and feelings, and ability to discern between what is or is not real: P5 “With PTSD you learn you cannot trust your feelings … the uncertainty,” P6 “What disturbed me is that the feeling was actually very familiar […] you always question: is this normal? Do I actually experience this stuff? Is everybody experiencing this?” One participant explicitly reported that the feeling experienced during the RHI closely resembled the dissociative experience: “It is a perfect illustration of when you feel and not feel a sensation […] when you feel it and then don't feel it in connection with your body […] that's the experience when you are really … in the middle of something.”

#### Body ownership and sense of agency

Finally, one participant described the correlation between body awareness and the body in movement: P6 “If I don't use a part [of my body] I am not aware of it. I am aware of them when I use them,” where moving the body (sense of agency) appears to support the sense of body ownership. Also, not being allowed to move the hands during the experiment alters the ability to identify and respond to feelings of disconnection from parts of the body: P4 “I couldn't fix what was happening,” P3 “It was hard to stay still when you were doing it,” P2 [asked what makes it difficult to stay present] “Staying in one position.”

## Discussion

The aim of this study was to investigate sense of body ownership and its relation to sense of agency in PTSD and its dissociative subtype (PTSD+DS) through manipulation of multisensory integration processes via the RHI paradigm. As expected, the results of the study revealed an overall stronger effect of the illusion during the synchronous as compared to the asynchronous condition, measured by both proprioceptive drift and by subjective ratings on the perception of the illusion. During the synchronous condition, the PTSD group showed a significantly *lower* effect of the illusion as compared to the healthy control group, indicated by lower proprioceptive drift and subjective rating on the perception of the illusion. By contrast, the PTSD+DS group exhibited a high variance in response to the RHI, ranging from very strong to very weak, both in terms of proprioceptive drift and the subjective perception of the illusion. Moreover, the results showed a trend indicating that the lower the sense of agency, the stronger the effect of the RHI, as measured by subjective ratings of the illusory perception in PTSD.

Despite a small sample size, these results nonetheless suggest a pattern of response to manipulation of body representation in the PTSD group. Specifically, overall PTSD participants showed a very small illusion effect (see Figures 2, 3 for proprioceptive drift and subjective ratings) that was significantly lower than that observed in the HC group. As previously indicated, typical symptoms of PTSD include effortful avoidance of trauma-related distress as well as emotional numbing represented in mind and body (Frewen et al., 2012; APA, 2013). In PTSD, the top-down representation of the body, responsible in part for cognitive control, may predominate, filtering and suppressing sensory information that can lead to the manifestation of other typical PTSD symptoms, such as re-experiencing and hyperarousal. Indeed, the data captured here suggest that, overall, PTSD participants may have resorted to avoidant coping strategies in an attempt to maintain control of the body, reacting to sensory manipulation with a sustained rigid body image, which comprises perceptions, beliefs, and emotional representations relative to one's own body (Costantini and Haggard, 2007; de Vignemont, 2011).

By contrast, the PTSD+DS group displayed a highly variant response to the RHI, both in terms of proprioceptive drift and of subjective perception of the illusion. Phenomenological reports suggest that the conflict between top-down representation of the body and bottom-up sensory information was a familiar feeling to these participants. They described becoming uncertain about the reality of their perceptions and/or the quality of their body representation. Broadly speaking, two coping/defensive strategies were observed. The first strategy involved the individual reacting to the presumed unresolved conflict between top-down representation of the body and bottom-up sensory information with depersonalization, where both the sense of agency and the sense of ownership were reported to be affected. These individuals reported experiencing both detachment from the body or parts of the body (an extreme example represented by out-of-body experiences) and/or freezing responses during which he/she was unable to move parts of his/her body (Bracha, 2004; Schauer and Elbert, 2010; Panksepp and Biven, 2012; Ataria, 2015; Frewen and Lanius, 2015). Such freezing responses have been proposed to involve thalamocortical deafferentiation, where bottom-up sensory signals no longer influence higher cortical regions mediating integration of the experience (Longo et al., 2008; Lanius et al., 2014), a reaction also observed in animal models under threat (Kalin et al., 2005; Mobbs et al., 2009; Porges, 2009; Kozlowska et al., 2015). For example, one PTSD+DS participant who experienced freezing of the hand during the RHI reported the following sensation at the end of the experimental session: “Feeling tingling, like wearing a glove… like when I'm freezing and then the sensation comes back.” We hypothesize that severely traumatized individuals would resort to this strategy as an extreme defense to a potential threat, when all other coping strategies (e.g., avoidance) are unavailable or unhelpful (Herman, 1992), with consequent drifting toward a dissociative state involving depersonalization and derealization.

The second defensive strategy to cope with the presumed conflict between top-down representation of the body and bottom-up sensory information observed in the PTSD+DS group was similar to the strategy proposed for the PTSD group. Here, top-down cognitive representation dominated, thus having the potential to suppress afferent signals in order to maintain control over the body, body ownership and sense of agency. Given the high variance characterizing the response in the PTSD+DS group, our results suggest that patterns of response to the manipulation of body ownership in the dissociative subtype of PTSD may be critically dependent on an individual's state at the time of testing, which can change over time and which is characterized by alterations in integrating multisensory information. Here, it is also interesting to note that previous neuroimaging studies of dissociative states in PTSD involving depersonalization have suggested altered activity in brain regions involved in multisensory integration during states of depersonalization/derealization (Simeon et al., 2000; Lanius et al., 2002; Felmingham et al., 2008). Future research examining the RHI illusion at multiple time points will therefore be of utmost importance.

Taken together, these results support the notion that high-level cortical processes (as interpretation of experienced body-related event) can modulate low-level subcortical mechanisms such as multisensory integration. Here, psychiatric symptoms originating from traumatic events would affect not only the psychological domain, but also somatic processes (Eshkevari et al., 2012; Walsh et al., 2015), with effects on embodiment and body ownership partially resembling those demonstrated previously in neurological patients with somatosensory system lesions (Lenggenhager et al., 2012).

Finally, these results point toward a close interrelation between sense of agency and sense of body ownership (in terms of subjective perception of the illusion) in PTSD. Specifically, a weaker sense of agency (measured here as a continuous feeling of being in control of one's body movements; Haggard and Chambon, 2012) showed a trend toward a significant correlation with a stronger perception of the illusion. Participants' self-reports were in line with this observation where they described utilizing intentioned movement as a means to reinforce sense of agency when they began to perceive that they were losing their sense of body ownership. Critically, severe dissociative symptoms have been associated with the loss of either body ownership and sense of agency (Ataria, 2015, 2016).

Limitations of the current study need to be considered along with the conclusions. Firstly, the small sample size within the three groups does not allow for generalization of the results. Further investigation in larger samples is required. Secondly, data were collected at a single time point, whereas a longitudinal design would allow for investigation of differential psychological/physiological states in PTSD+DS. Moreover, the different location of the control group recruitment and data collection might represent a confounding variable, although experimental protocols have been accurately compared and followed. Finally, the RHI protocol did not include directly procedures to manipulate sense of agency. Future studies investigating the RHI in PTSD should include manipulation of either sense of agency and sense of body ownership in order to explore the impact of each independently and in combination. In addition, future studies should enrich behavioral observations with physiological and neuroimaging data to delineate the neurophenomenology of body ownership and sense of agency in PTSD and its dissociative subtype.

In conclusion, this study contributes to a deeper understanding of the complex defensive reaction occurring during manipulation of body ownership in traumatized individuals with PTSD and its dissociative subtype. Furthermore, our results highlight key differences in patterns of response to the RHI between the two groups. Whereas a top-down filtering of sensory information as a cognitive avoidance strategy aimed at maintenance of a rigid body representation may characterize the PTSD group, a changing state-dependent representation of the body appears to better describe individuals with PTSD+DS. Crucially, sense of agency is thought to play a primary role in the maintenance and recovery of body ownership in PTSD. Indeed, our findings showed that a lower sense of agency correlated with a stronger illusion effect, with PTSD individuals resorting frequently to intentional movements in order to regain a sense of body ownership during dissociative experiences. Taken together, these findings point toward the need for development of specific treatments for the dissociative subtype of PTSD that are tailored to address not only alterations in body representation but also potential loss of body ownership. Interventions that focus on increasing the feeling of connection with one's own body such as body scan mindfulness training (Lanius et al., 2015; Boyd et al., 2017) or sensorimotor psychotherapy (Ogden and Fisher, 2014; Frewen and Lanius, 2015) may be helpful in this regard. Finally, improving and restoring an embodied sense of agency may be critical to trauma recovery.

## Author contributions

DR, PF, and RL: conception and study design; DR, DB, SH, CL, and RL: study execution and supervision; DR, DB, PF, MM, and RL: data analysis and interpretation; DR, DB, SH, CL, PF, MM, and RL: manuscript drafting and revision.

### Conflict of interest statement

The authors declare that the research was conducted in the absence of any commercial or financial relationships that could be construed as a potential conflict of interest.

## Footnotes

^1^Depending on time of enrolment.
