# Supplementary material for: Altered Sense of Body Ownership and Agency in Posttraumatic Stress Disorder and Its Dissociative Subtype: A Rubber Hand Illusion Study
Source: Front Hum Neurosci. 2018 May 1;12:163. doi: 10.3389/fnhum.2018.00163 (PMC5938392; doi:10.3389/fnhum.2018.00163)
Supplement: Supplementary file 1 [file Data_Sheet_1.DOCX]

**Participant ID________________ Date________________________**

**Order: Asynch Synch**

**Questionnaire on the Rubber Hand Illusion**

(Botvinick M, Cohen J (1998). Rubber hands 'feel' touch that eyes see. *Nature*, 391)

Please indicate your level of agreement with each statement by drawing a mark on each continuous line. The left extreme indicates complete disagreement (- 3) and the right extreme indicated complete agreement (+3).

**During the experiment there were times when:**

1. It seemed as if I were feeling the touch of the paintbrush in the location where I saw the rubber hand touched.

2. It seemed as though the touch I felt was caused by the paintbrush touching the rubber hand.

3. I felt as if the rubber hand were my hand.

4. It felt as if my (real) hand were drifting towards the rubber hand.

5. It seemed as if I might have more than one right hand or arm.

6. It seemed as if the touch I was feeling came from somewhere between my own hand and the rubber hand.

7. It felt as if my (real) hand were turning ‘rubbery’.

8. It appeared (visually) as if the rubber hand were drifting towards my hand.

9. The rubber hand began to resemble my own (real) hand, in terms of shape, skin tone, freckles or some other visual feature.
