# Supplementary material for: Altered Sense of Body Ownership and Agency in Posttraumatic Stress Disorder and Its Dissociative Subtype: A Rubber Hand Illusion Study
Source: Front Hum Neurosci. 2018 May 1;12:163. doi: 10.3389/fnhum.2018.00163 (PMC5938392; doi:10.3389/fnhum.2018.00163)
Supplement: Supplementary file 2 [file Data_Sheet_2.DOCX]

**Participant ID________________ Date________________________**

**Questionnaire on the Rubber Hand Illusion- Trait Sense of Agency**

(Kalckert & Ehrsson, 2012 edited version)

Please indicate your level of agreement with each statement by drawing a mark on each continuous line. The left extreme indicates complete disagreement (- 3) and the right extreme indicated complete agreement (+3). Please note that the following questions will focus on how you have been feeling with respect to your body **during the last month**. When answering, if you have been experiencing particular feelings relative to parts of your body (e.g. hands, feet, head), please consider these body parts when giving your score.

**Usually (during the last month)…**

1) ...My body/part of my body moves just like I want to, as if it obeys my will.

2) …I feel as if I can control the movements of my body/part of my body.

3) … I feel as if I cause the movement of my body/part of my body.

4) … I feel as if my body is controlling my will.

5) … I can sense my movements from somewhere else with respect to my body/part of my body.

6) … It seems as if my body/part of my body has a will of its own.

7) If you were referring to “part of my body” while giving your answer, please specify what part of your body __________________________________________________________________
